# Registro do Sul do Brasil de Fibrilação Atrial (SBR-AF): Preditores de Recorrência de Arritmia Atrial Após a Primeira Ablação por Cateter

**DOI:** 10.36660/abc.20240871

**Published:** 2025-02-04

**Authors:** Muhieddine Omar Chokr

**Affiliations:** 1 Rede D’Or de São Paulo São Paulo SP Brasil Rede D’Or de São Paulo, São Paulo, SP – Brasil; 2 Hospital das Clínicas da Faculdade de Medicina da Universidade de São Paulo Instituto do Coração São Paulo SP Brasil Instituto do Coração do Hospital das Clínicas da Faculdade de Medicina da Universidade de São Paulo, São Paulo, SP – Brasil

**Keywords:** Eletrofisiologia, Fibrilação Atrial, Sistema de Registros, Brasil

A fibrilação atrial (FA) tem se consolidado como um problema de saúde pública, afetando até 4% da população. Os pacientes acometidos apresentam aumento nas taxas de mortalidade, acidente vascular cerebral e insuficiência cardíaca, bem como maior número de internações e piora na qualidade de vida.^1^

O tratamento moderno dessa arritmia inclui o controle dos fatores de risco cardiovasculares, a prevenção de eventos cardioembólicos e o controle do ritmo cardíaco. Atualmente, entre as diferentes possibilidades terapêuticas, a ablação por radiofrequência se estabeleceu como a terapia de eleição, apresentando os melhores resultados no manejo dessa condição clínica. Na forma paroxística da FA, gatilhos provenientes das veias pulmonares são, na maioria dos casos, os responsáveis por deflagrar a arritmia. Por outro lado, na forma persistente, diferentes mecanismos eletrofisiológicos estão envolvidos, tornando a estratégia de tratamento invasivo mais complexa com o aumento do tempo de evolução da doença.^2^

De acordo com as diretrizes mais recentes da Sociedade Europeia de Cardiologia (ESC, 2020), a principal indicação para a adoção de uma estratégia de controle do ritmo é a redução dos sintomas relacionados à FA e a melhora na qualidade de vida dos pacientes. Contudo, o estudo EAST-AFNET 4 demonstrou que uma estratégia precoce de controle do ritmo pode reduzir significativamente o risco de desfechos cardiovasculares graves em comparação com os cuidados habituais, incluindo a redução da mortalidade em subgrupos específicos. Esse achado pode redefinir a abordagem no manejo da FA.^1,3^

Embora os resultados da ablação tenham melhorado ao longo dos últimos anos com a evolução tecnológica, eles ainda são heterogêneos em diferentes regiões do mundo, variando de acordo com os recursos tecnológicos disponíveis e a experiência dos grupos envolvidos.

Nesse contexto, várias sociedades ao redor do mundo têm se organizado para criar registros de dados de mundo real, com o objetivo de documentar tanto o sucesso quanto as complicações associadas à terapia intervencionista para a FA. Essas iniciativas constituem a base para a gestão da qualidade e a identificação de possíveis melhorias nos procedimentos de ablação por cateter. Embora ambiciosa, essa abordagem é essencial e impacta diretamente a indicação do procedimento, além de influenciar positivamente a qualidade de vida dos pacientes submetidos à intervenção.^4^

Nesta edição dos Arquivos Brasileiros de Cardiologia, os autores apresentam o Registro do Sul do Brasil de Fibrilação Atrial^5^ de longo prazo que demonstra ser possível alcançar resultados semelhantes aos obtidos em países desenvolvidos. O trabalho destaca a importância do tratamento precoce na redução das recorrências de FA.

O registro é notável pelo longo período de acompanhamento de mais de mil pacientes submetidos à ablação de FA, fornecendo dados de mundo real em uma região onde informações em grandes populações ainda eram escassas. Essas informações são fundamentais para que o cardiologista responsável pela recomendação do procedimento possa basear-se em dados nacionais e não apenas em evidências externas.

Os autores relatam uma taxa de recorrência de 18,6% na forma paroxística e de 29,8% na forma persistente da FA após um único procedimento, além de demonstrar que o risco de recorrência diminui após o primeiro ano de acompanhamento. A taxa de complicações relacionadas à intervenção foi de 2,1%, um dado relevante para o cardiologista clínico que realiza o acompanhamento ambulatorial do paciente, conferindo segurança ao recomendar o procedimento, uma vez que os índices são comparáveis aos dos grandes centros mundiais.^4^

Os autores merecem elogios por apresentarem dados em larga escala do mundo real na América Latina, demonstrando que é possível alcançar, por meio da ablação por cateter, resultados semelhantes aos observados em países de alta renda.

Além disso, a organização do banco de dados com seguimento de longo prazo deve servir de exemplo para outros grupos nacionais, incentivando a documentação dos resultados agudos e de longo prazo da ablação. Essa prática pode demonstrar que os resultados obtidos são semelhantes em diferentes regiões da América Latina, funcionando como uma métrica de qualidade da intervenção realizada. Por fim, a introdução dinâmica de novas tecnologias no arsenal terapêutico do eletrofisiologista, pode impactar positivamente os resultados obtidos, tornando essencial o acompanhamento contínuo e detalhado desses dados.
