# Supplementary material for: Registro do Sul do Brasil de Fibrilação Atrial (SBR-AF): Preditores de Recorrência de Arritmia Atrial Após a Primeira Ablação por Cateter
Source: Arq Bras Cardiol. 2025 Feb 4;122(1):e20240871. [Article in Portuguese] doi: 10.36660/abc.20240871 (PMC11809888; doi:10.36660/abc.20240871)
Supplement: Translation

# Translation: Southern Brazil Registry of Atrial Fibrillation (SBR-AF): Predictors of Atrial Arrhythmia Recurrence After First Catheter Ablation

**DOI:** 10.36660/abc.20240871i

**Authors:** Muhieddine Omar Chokr

**Affiliations:** 1 São Paulo SP Brazil Rede D’Or de São Paulo, São Paulo, SP – Brazil; 2 São Paulo SP Brazil Instituto do Coração do Hospital das Clínicas da Faculdade de Medicina da Universidade de São Paulo, São Paulo, SP – Brazil

**Keywords:** Electrophysiology, Atrial Fibrillation, Registries, Brazil

Atrial fibrillation (AF) has established itself as a significant public health issue, affecting up to 4% of the population. Patients with AF experience increased mortality rates, stroke, and heart failure, as well as higher hospitalization rates and reduced quality of life.^1^

Modern treatment of this arrhythmia involves managing cardiovascular risk factors, preventing cardioembolic events, and controlling heart rhythm. Among the therapeutic options, radiofrequency ablation has established itself as the therapy of choice, presenting the best results in the management of this clinical condition. In paroxysmal AF, triggers originating from the pulmonary veins are primarily responsible for initiating the arrhythmia. On the other hand, in the persistent form, different electrophysiological mechanisms are involved, making the invasive treatment strategy more complex as the disease progresses over time.^2^

According to the latest guidelines from the European Society of Cardiology (ESC, 2020), the primary indication for rhythm-control strategies is to alleviate AF-related symptoms and improve the patient's quality of life. However, the EAST-AFNET4 trial demonstrated that early rhythm-control strategies significantly reduce the risk of severe cardiovascular outcomes compared to usual care, including reduced mortality in specific subgroups. This finding may redefine approaches to AF management.^1,3^

Although the results of ablation have improved over the past years with technological advancements, they remain heterogeneous in different regions of the world, varying according to the available technological resources and the experience of the involved teams.

In this context, various societies around the world have organized to create real-world data registries with the aim of documenting both the success and the complications associated with interventional therapy for AF. These initiatives form the foundation for quality management and the identification of potential improvements in catheter ablation procedures. Although ambitious, this approach is essential and directly impacts the indication for the procedure, as well as positively influencing the quality of life of patients undergoing the intervention.^4^

In this edition of *Arquivos Brasileiros de Cardiologia*, the authors present the Southern Brazil Registry of Atrial Fibrillation (SBR-AF),^5^ a long-term Brazilian registry demonstrating that outcomes similar to those in developed countries can be achieved. The study highlights the importance of early treatment in reducing AF recurrences.

The registry is notable for its long follow-up period involving over a thousand patients who underwent AF ablation, providing real-world data in a region where information on large populations was previously scarce. This information is essential for cardiologists responsible for recommending the procedure, enabling them to rely on national data rather than exclusively on external evidence.

The authors report a recurrence rate of 18.6% in the paroxysmal form and 29.8% in the persistent form of AF after a single procedure, in addition to demonstrating that the risk of recurrence decreases after the first year of follow-up. The complication rate related to the intervention was 2.1%, a relevant finding for the clinical cardiologist managing the patient in outpatient care, providing confidence in recommending the procedure, as these rates are comparable to those of major global centers.^4^

The authors deserve commendation for presenting large-scale real-world data from Latin America, demonstrating that it is possible to achieve outcomes through catheter ablation comparable to those observed in high-income countries.

Furthermore, the organization of the database with long-term follow-up should serve as an example for other national groups, encouraging the documentation of both acute and long-term ablation outcomes. This practice can demonstrate that the results achieved are consistent across different regions of Latin America, serving as a quality metric for the intervention performed. Finally, the dynamic introduction of new technologies into the electrophysiologist's therapeutic arsenal can positively impact the outcomes achieved, making continuous and detailed monitoring of these data essential.
